# Effects of sacubitril-valsartan on central and obstructive apneas in heart failure patients with reduced ejection fraction

**DOI:** 10.1007/s11325-022-02623-0

**Published:** 2022-04-29

**Authors:** Youmeng Wang, Roberto Fernandes Branco, Matthew Salanitro, Thomas Penzel, Christoph Schöbel

**Affiliations:** 1 Sleep Medicine Center, Charité-Universitätsmedizin, Charitéplatz 1, 10117 Berlin, Germany; 2 Universitätsmedizin Essen, Ruhrlandklinik - Westdeutsches Lungenzentrum, am Universitätsklinikum Essen GmbH, Tüschener Weg 40, 45239 Essen, Germany

**Keywords:** Heart failure, Sacubitril-valsartan, ARNI, Central apneas, Obstructive apneas

## Abstract

**Objective:**

This study aimed to evaluate the effect of sacubitril-valsartan (SV) on central apneas (CA) and obstructive apneas (OA) in patients with heart failure with reduced ejection fraction (HFrEF).

**Methods:**

In patients with HFrEF, SV initiation was titrated to the highest tolerable dosage. Patients were evaluated with portable apnea monitoring, echocardiography, and cardiopulmonary exercise testing at baseline and 3 months later.

**Results:**

Of a total of 18 patients, 9 (50%) had OA, 7 (39%) had CA, and 2 (11%) had normal breathing. SV therapy was related to a reduction in NT-pro BNP and an improvement in LV function after 3 months. Portable apnea monitoring revealed a significant decrease of the respiratory event index (REI) after treatment with SV (20 ± 23 events/h to 7 ± 7 events/h, *p* = 0.003). When subgrouping according to type of apneas, REI, and time spent below 90% saturation (T90) decreased in patients with CA and OA (all *p* < 0.05).

**Conclusion:**

In this prospective study, SV treatment for 3 months in patients with CA and OA is associated with a significant decrease in REI.

## Introduction

Heart failure (HF) is now recognized as a severe health issue affecting almost 65 million people of all ages worldwide. The prevalence of HF is 1–2% in patients over 65 years old, and it appears to be increasing in developed countries [1]. Despite substantial breakthroughs in medical and surgical treatment of HF, approximately 30% of patients are admitted annually for HF exacerbation [2]. Central apneas (CA) and obstructive apneas (OA) are increasingly recognized comorbidity in subjects with HF and may affect the prognosis of HF [3]. To date, there is consensus that the initial step in managing patients with CA/OA and HF should be optimizing HF treatment [4]. Indeed, past research has shown that optimizing pharmacological therapy [5, 6] and utilizing non-pharmacological ways to treat HF can improve CA/OA [7]. However, the best way to manage CA/OA in HF is still being debated, owing to the fact that the therapeutic benefit of additional respiration treatment for patients with HFrEF has been questioned following the SERVE-HF trial’s results and ongoing findings of the ADVENT-HF research, respectively. The results showed that not only was adaptive servo-ventilation (ASV) ineffective, but also a post hoc analysis found excessive cardiovascular mortality in patients who received the treatment [8].

Sacubitril-valsartan (SV) is a first-in-class angiotensin-receptor neprilysin inhibitor used to treat HFrEF (New York Heart Association [NYHA] functional class II–IV) [9]. Therapy with SV decreased cardiovascular death, overall mortality, and HF-related hospitalizations in the PARADIGM-HF study compared to treatment with enalapril [10]. In preliminary investigations, angiotensin-converting enzyme (ACE) inhibitors have been shown to ameliorate CA/OA in patients with HF [11]. Despite the fact that the combination therapy can improve apneas in patients with HF, there is little research on the effect of SV on CA/OA [12]. In this study, we investigated the effect of initiating SV on apneas and hypothesized that CA/OA would improve when using treatment with SV.

## Methods

### Study population

This trial was a 3-month, single-center, open-label, prospective study from January 2019 to July 2021. Inclusion criteria were as follows: non-childbearing female and male patients age 60 + with HF (NYHA class II–IV); LVEF ≤ 40%; patients had to receive stable doses (at least 1 month) standard-of-care HF medication before the study; a blood test result of serum potassium ≤ 5.2 mmol/L, estimated glomerular filtration rate (eGFR) ≥ 30 ml/min/1.73 m^2^, and systolic blood pressure (SBP) ≥ 100 mmHg. Exclusion criteria were as follows: severe valvular disease, isolated right HF, secondary cardiomyopathy, hypertrophic obstructive cardiomyopathy, previous or upcoming heart transplantation, and unstable angina within half a year before the study; patients treated with a history of angioedema or significantly increased liver enzymes (at least three times higher than the upper threshold), or with combination drugs such as ACE inhibitors and angiotensin-receptor blockers (ARBs). To participate in this study, the subjects were required to provide written informed permission. Our study was registered with ClinicalTrials.gov, number NCT02768298, and the EU Clinical Trials Register, number CLCZ696BDE01.

### Study drug

According to the dosage approved by European Union, patients took SV twice a day and adjusted it for renal function and hemodynamic tolerance. Patients were advised to take the study drug simultaneously every day, according to the approved instructions that follow the current European HF guidelines’ best medical treatment recommendations.

### Home portable apnea monitoring

The ApneaLink device (ResMed Inc., Martinsried, Germany) was used to measure nasal flow and pulse oximetry in this study [13]. Participants were instructed to use the device in a standardized manner by study personnel who had undergone extensive training. Adults with apnea can be assessed using portable apnea monitoring devices instead of overnight polysomnography [14, 15]. Apnea was defined as a reduction in airflow of more than 90% from baseline for more than 10 s. Apneas were further classified as OA if there was any evidence of respiratory effort, CA if there was no evidence of respiratory effort, and mixed apnea if features of both CA and OA were present. For the purposes of this study, hypopnea was described as a 30% decrease in airflow lasting for more than than 10 s, followed by a 3% reduction in oxygen saturation. The number of apnea and hypopnea events per hour of monitoring during a certain period was described as the respiratory event index (REI). The REI is used as a surrogate for the apnea–hypopnea index (AHI) because it measures time spent monitoring rather than total sleep time [16].

The changes in echocardiographic parameters from baseline were examined in patients with HF who had a baseline LVEF of less than 40%. A ramp technique was used following calibration on a treadmill, and a cardiopulmonary exercise test (CPET) was performed on the patients after taking their age and gender into consideration [17]. Normative clinical chemistry tests were performed which included a full blood count and the N-terminal segment of the pro-brain natriuretic peptide (NT-pro BNP). These procedures were supervised and managed by a clinically experienced cardiologist and nurse.

### Statistical analysis

Descriptive data are presented as means ± standard deviation (SD) or as numbers and percentages of each category unless otherwise indicated. Paired *t*-tests (for data with normal distribution) and Wilcoxon tests (for data with abnormal distribution) were used due to the reliance of both populations before and after. The level of statistical significance was established at *p* < 0.05. All statistical data were performed using SPSS version 25.0 (IBM SPSS Statistics, Armonk, NY, USA).

## Results

A total of eighteen eligible patients were enrolled in the study. Table 1 summarizes the clinical, demographic, and medications data. Despite being given optimal medical treatment, most subjects had apneas at baseline. Only 2 patients (11%) had normal breathing, 9 had OA (50%), and 7 had CA (39%). Among subjects with OA, 4 (23%), 5 (27%), and 0 (0%) had mild (5 ≤ REI < 15), moderate (15 ≤ REI < 30), and severe (REI ≥ 30) apnea, respectively, while among subjects with CA, 4 (22%), 0 (0%), and 3 (17%) had mild, moderate, and severe apnea, respectively. Before using the ApneaLink monitoring, the patients were requested to stop taking any medications that had a direct impact on ventilatory control.

**Table 1:** Baseline characteristics of patients

HF patients treated with SV (*n* = 18)	
Age (years)	66.7 ± 10.7
Gender (male/female, *n*)	15/3
BMI (kg/m^2^)	43.8 ± 50.2
NYHA class (%)	
Class II	50
Class III	50
Atrial fibrillation (%)	22
CKD (%)	39
Diabetes (%)	17
Hypertension (%)	78
COPD (%)	28
Cardiac infarction (%)	22
Beta-blocker (%)	89
Loop diuretics (%)	72
ICD (%)	72
CRT (%)	17

*BMI* body mass index; *COPD* chronic obstructive pulmonary disease; *CKD* chronic kidney disease; *CRT* cardiac resynchronization therapy; *ICD* implantable cardioverter defibrillator; *NYHA* New York Heart Association

**Fig. 1:**
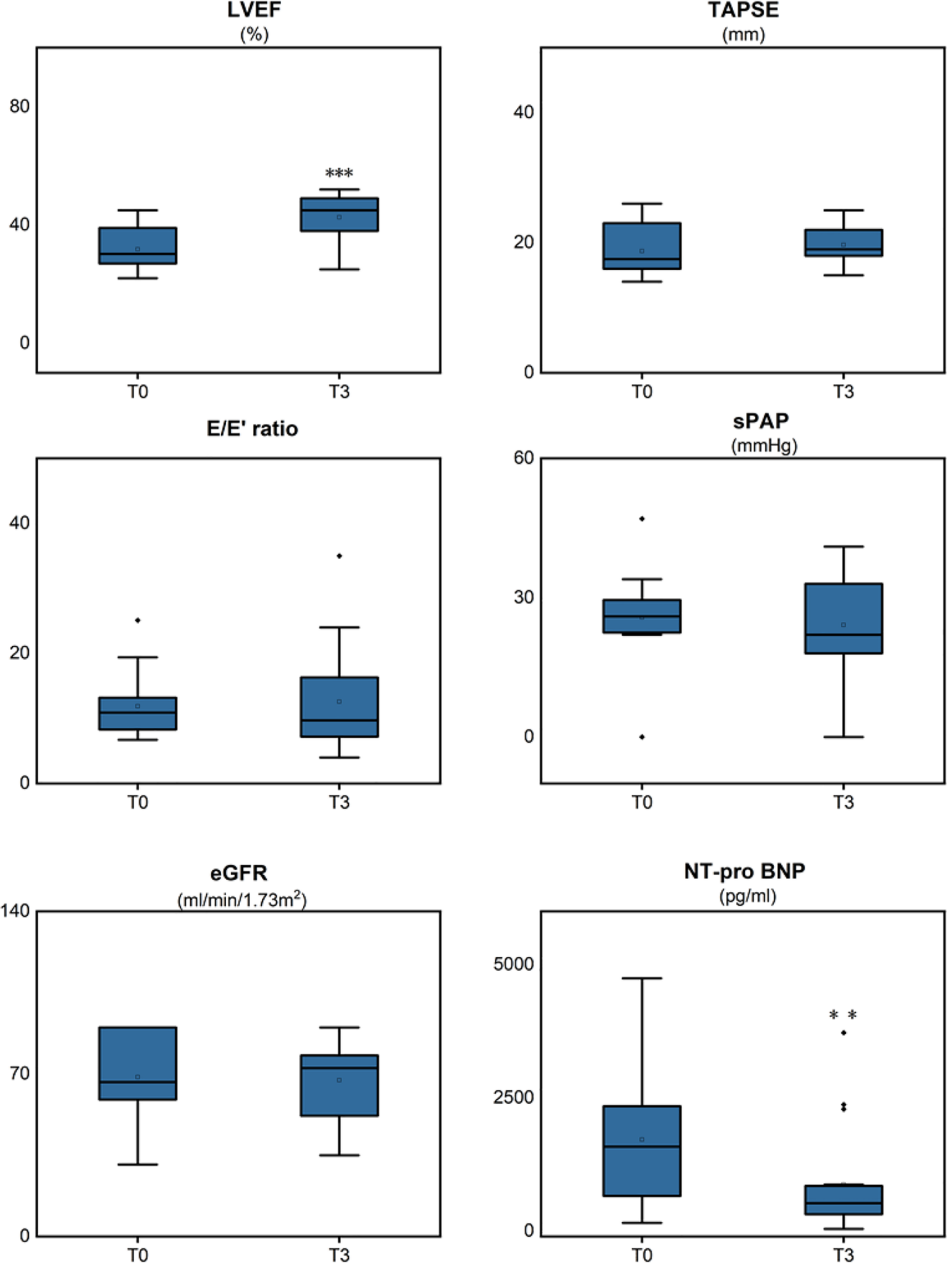
Changes in echocardiographic measures and blood examination after 3 months of treatment with SV. *p < 0.05; **p < 0.01; ***p < 0.001. eGFR estimated glomerular filtration rate; LVEF left ventricular ejection fraction; NT-proBNP pro-B-type natriuretic peptide; sPAP systolic pulmonary artery pressure; TAPSE tricuspid annular plane systolic excursion

Results of SV on cardiac function, CPET, and blood testing in the overall population are presented in Table 2 and Fig. 1*.* SV has been shown to be associated with a statistically significant decrease in NT-pro BNP. The administration of the drug was also associated with improved left ventricular (LV) systolic and diastolic function, as indicated by an increase in LV end-diastolic diameter, as well as with improvement in LV reverse remodeling, as indicated by increased LVEF. No statistically significant changes were noted in tricuspid annular plane systolic excursion (TAPSE) and systolic pulmonary artery pressure (sPAP). There were no differences in peak oxygen consumption or FEV_1_ (both *p* > 0.05) after therapy at CPET compared to baseline.

**Table 2:** Relation between SV treatment and heart remodeling, CPT, and blood examination

	Baseline	3 months	*p*-value
LVEF (%)	32 ± 7	43 ± 9	< 0.001a
LVEDD (mm)	59.9 ± 6.9	56.8 ± 9.6	0.025a
LVESD (mm)	50.7 ± 9.1	44.5 ± 10	0.001a
LVESV (ml)	110.4 ± 48.1	83.3 ± 39.7	0.001b
TAPSE (mm)	18.7 ± 4.2	19.7 ± 2.9	0.392b
E/E'	11.9 ± 4.9	12.6 ± 7.8	0.594a
sPAP (mmHg)	25.8 ± 10.7	24.1 ± 10.8	0.623b
FEV_1_ (L)	2.6 ± 1	2.9 ± 0.8	0.209a
Max VO_2_ (ml/min/kg)	14.4 ± 2.3	13.8 ± 2.4	0.296a
eGFR (ml/min/1.73 m^2^)	68.8 ± 17.8	67.3 ± 16.6	0.576a
NT-pro BNP (pg/ml)	1792.1 ± 1271.3	876.9 ± 984.2	0.001b

*eGFR* estimated glomerular filtration rate; *FEV*_*1*_ forced expiratory volume for one second; *LVEF* left ventricular ejection fraction; *LVEDD* left ventricular end-diastolic diameter; *LVESD* left ventricular end-systolic diameter; *LVESV* left ventricular end-systolic volume; *NT-proBNP* pro-B-type natriuretic peptide; *RV-FAC* right-ventricular fractional area change; *sPAP* systolic pulmonary artery pressure; *TAPSE* tricuspid annular plane systolic excursion; *VO*_*2*_ oxygen consumption; *P*^*a*^ represents the paired *T*-test; *P*^*b*^ represents Wilcoxon test

**Fig. 2:**
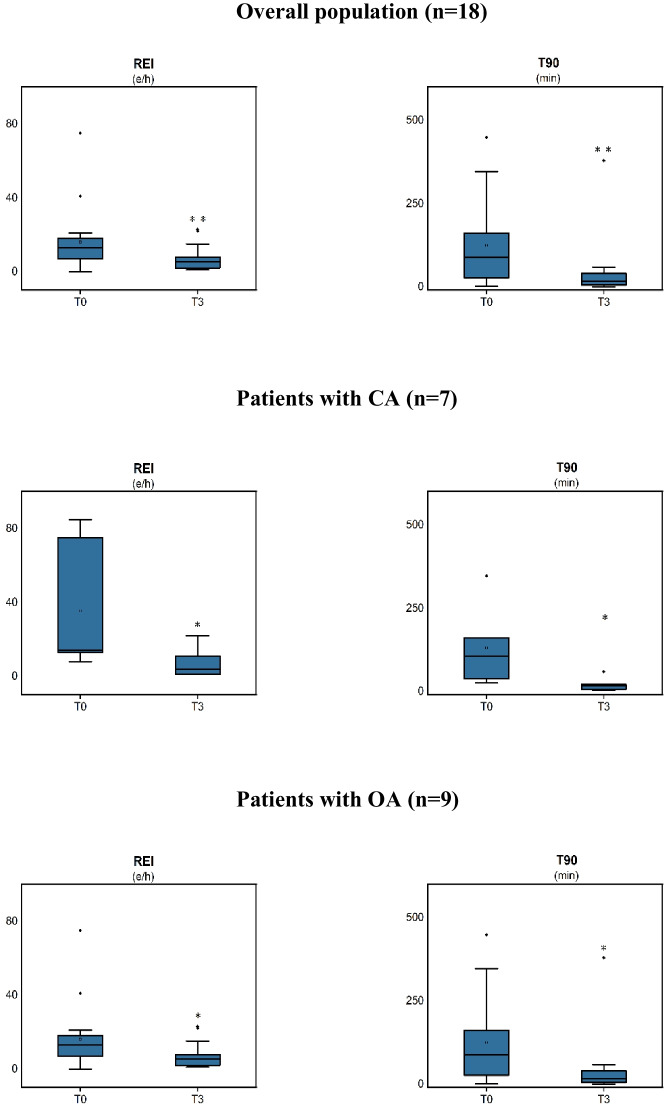
Changes of REI and T90 after 3-month SV treatment in the overall population and in the subgroups with OA and with CA. *p < 0.05; **p < 0.01; ***p < 0.001. CA central apnea; OA obstructive apnea; REI respiratory event index; T90 time spent with oxygen saturation < 90%

SV treatment was found to be associated with a significant reduction in REI in the general population (Table 3 and Fig. 2). The effect of SV administration was significantly associated with a decrease in REI in the subgroup of subjects with OA (by 47%). In the subgroup of subjects with CA, SV was also associated with a decrease in REI (by 81%). SV had a decreasing effect on the minimal oxygen saturation and T90% (all *p* < 0.05).

**Table 3:** Relation between SV treatment and apneas

	Baseline	3 months	p-value
Overall population (*n* = 18)			
REI (e/h)	20 ± 23	7 ± 7	0.003^b^
SaO_2_ basal (%)	93 ± 2	95 ± 2	0.053^a^
SaO_2_ min (%)	80 ± 4	80 ± 8	0.812^a^
T90 (min)	119 ± 128	42 ± 86	0.001^b^
Patients with CA (*n* = 7)			
REI (e/h)	36 ± 32	7 ± 8	0.018^b^
SaO_2_ basal (%)	94 ± 2	94 ± 2	0.876^a^
SaO_2_ min (%)	79 ± 4	77 ± 11	0.598^a^
T90 (min)	131 ± 117	19 ± 19	0.028^b^
Patients with OA (*n* = 9)			
REI (e/h)	14 ± 6	7 ± 7	0.039^a^
SaO_2_ basal (%)	92 ± 3	95 ± 2	0.025^a^
SaO_2_ min (%)	81 ± 2	82 ± 6	0.404^b^
T90 (min)	138 ± 151	66 ± 119	0.038^b^

*CA* central apnea; *OA* obstructive apnea; *REI* respiratory event index; *SaO*_*2*_ oxygen saturation; *T90* time spent with oxygen saturation < 90%; *P*^*a*^ represent the paired *T* test; *P*^*b*^ represent Wilcoxon test

## Discussion

SV has been shown to benefit both CA and OA in patients with HFrEF. The administration of SV to optimal medical therapy was associated with a significant decrease in the REI.

A comparison of SV and enalapril has shown that the former was more effective at decreasing all-cause and sudden-death mortality, as well as limiting the progression of HF [18]. This study shows that SV is associated with an increase in LVEF, which in turn promotes LV and left atrial reverse remodeling and an improvement in REI [12]. As expected, SV also had a positive effect on NT-pro BNP [19, 20]. It is worth noting that some participants transitioned from CA to OA following therapy with SV, which consequently became the most common respiratory disorder. The administration of SV reduced CA, confirming the beneficial effect of the medication on CA stated previously in a previous case study [21]. In this study, successful cardiac function optimization by SV was related to a shift in the apnea phenotype from CA to OA. This finding is consistent with earlier studies, which have shown that improvements in cardiac performance lead to reduced CA, consequently unmasking previously undiagnosed OA [22–25]. Fox et al. found a 71-year-old man who suffered from HF and sleep-disordered breathing (SDB). Treatment with SV was associated with improved cardiac function, as measured by a decrease in NT-pro BNP and an increase in LVEF. This was associated with a significant decrease in the AHI. This is the first case to demonstrate improvement in HF and SDB following the start of SV treatment [26].

SV, by inhibition of neprilysin, prevents the degradation of natriuretic peptides, hence enhancing their natriuretic and vasodilatory actions and lowering pulmonary congestion, respectively [27, 28]. Additionally, the beneficial effects on cardiac reverse remodeling, which are associated with enhanced LVEF, may increase cardiac output [29, 30]. Overall, those effects may promote effective ventilation and gas exchange, and the chemoreflex, which reduces pulmonary stretch receptor stimulation while increasing the perfusion of peripheral chemoreceptors [31]. Furthermore, an increase in cardiac output may decrease circulation time, reducing the amount of time available for the chemoreflex system to detect and respond to changes in CO_2_ [32]. Finally, the medication has been shown to reduce the amount of rostral fluid shift that occurs when a person is in a reclined position. Although the PARADIGM-HF trial made a small but significant contribution to improving survival, it is tempting to conclude that this can be attributed to the reduced apneic burden. It is equally tempting to consider SV as an alternative first-line therapeutic strategy for apneas and, specifically, CA in HF [10]. Additionally, there are more alternative therapeutic approaches for hypoxemic burden. Olaf et al. discovered that transvenous phrenic nerve stimulation (TPNS) could significantly reduce nocturnal hypoxemic load. Hypoxemic burden is more predictive of mortality than AHI and should be a primary indicator for CSA treatments [33]. However, to address these intriguing challenges precisely, larger cohorts with definitive outcomes followed for longer periods of time would be required.

### Limitations

This study has several limitations. First, we acknowledge this is a single-center study and requires further studies to support the generalizability of the findings presented. In addition, our study was limited to the older population with HFrEF. Another possible limitation is that portable monitoring devices do not record CO_2_ levels, sleep stages, and sleep position. As a result, conclusions concerning these factors cannot be drawn and events cannot be classified into different sleep stages. In addition, no electroencephalograms were recorded in this study. Thus, it was impossible to determine if patients were asleep during the assessment, which could underestimate the severity of OA and CA. Importantly, the ApneaLink may overestimate the REI, as actual sleep time may be shorter than recorded time, implying a higher prevalence and severity of apnea.

## Conclusion

In summary, our findings obtained from patients with HFrEF show that SV had positive effects on both CA and OA. The effects of SV are more limited on OA than CA. SV may become a promising therapeutic option for CA in HFrEF.

## Data Availability

The datasets generated and/or analyzed during the current study are available from the corresponding author on reasonable request.
